# Correlations of age and growth rate with microbiota composition in Atlantic cod (*Gadus morhua*) larvae

**DOI:** 10.1038/s41598-017-09073-9

**Published:** 2017-08-17

**Authors:** Ly T. T. Trinh, Ingrid Bakke, Olav Vadstein

**Affiliations:** 1 Department of Biotechnology and Food Science, NTNU Norwegian University of Science and Technology, N7491 Trondheim, Norway; 2 School of Biotechnology, International University, Vietnam National University, Quarter 6, Linh Trung ward, Thu Duc District, HoChiMinh City, Vietnam

## Abstract

Little information is available on the link between host development (growth rate and ontogeny) and the composition of the microbiota in fish larvae. This study was carried out to examine potential correlations of microbiota composition with age and growth rate of Atlantic cod larvae. Small and large cod larvae of the same age, representing slow and fast growing individuals, were sampled 10 times during a period of 42 days post hatching (dph), and the composition of the larval microbiota was investigated using a PCR/DGGE (Denaturing Gradient Gel Electrophoresis) strategy. We found significant differences in the intestinal microbiota of small and large larvae of the same age for 4 of the 10 age stages studied. We further found that the variation in the composition of the larval microbiota was more strongly correlated to age than to growth rate for larvae up to 28 dph, whereas for the older larvae growth rate and age was equally correlated to the composition of the microbiota. These results indicate that larval development may structure the microbiota through a change in selection pressure due to host-microbe and microbe-microbe interactions, and that the composition of the microbiota may influence larval development through improved energy gain.

## Introduction

The gastrointestinal tract (GI) of animals provides a habitat for a complex and diverse ecosystem of both aerobic and anaerobic microorganisms, and has been investigated by several groups during the last decade^1^. The intestinal microbiota plays an important role in many processes important for the viability of fish; including digestion and nutrition, and protection against the establishment of pathogenic microorganisms in the fish intestine^2–4^. Moreover, the gut microbiota significantly contributes in the normal development of fish, and under germfree conditions the development of a normal intestinal morphology and immune system maturation and function is impaired^5^.

Turnbaugh and colleagues^6^ demonstrated that the gut microbiota differed between obese and lean mice, and concluded that the gut microbiota contributed to obesity in mice by increasing the energy harvest from the diet. Also in humans the composition of the intestinal microbiota has been found to differ between obese and lean individuals, and the microbiota influences the efficiency of caloric extraction from the food^7^. Whereas obesity is a problem for humans, increased energy gain may be beneficial for farmed animals, especially in early life stages.

A wide range of microbes colonize the GI tract of fish, and they have been suggested to affect the growth and survival of the host^8, 9^. Forberg and coworkers^10^ showed that for both mangrove killifish and Atlantic cod, the composition of the larval microbiota differed between small and large individuals of the same age. However, their study was done only on one life stage. This suggests that there is a link between growth rate and composition of the microbiota in fish larvae. Based on the findings from mice^6, 7^ and fish^10^ we may hypothesize that the increased growth rate in some individuals may be explained by a microbiota providing increased ingestion, digestion and nutrition absorption for the host. A first step in testing this hypothesis is to see it the microbiota is different in slow and fast growing individuals.

Host-microbe interactions have a practical aspect for farmed animals, including fish, as they link to animal welfare and sustainability^11^. The aquaculture industry has grown considerably during the last decade and become a big protein producer. However, for most marine aquaculture species production of high quality juveniles is a major bottleneck, with the problem manifested as low growth rates, high mortality, malformation and low reproducibility during larval stages^8, 12, 13^. Currently, an increasing number of studies indicate that negative host-microbe interactions in fish larvae is a major cause of the observed problems^14–16^.

The main goal of this study was to investigate potential correlations between the composition of the microbiota and the growth rate of developing Atlantic Cod (*Gadus morhua*) larvae. We compared these possible differences with the succession of the microbiota taking place due to the ontogeny of the digestive tract. We used DGGE to characterize and compare the composition of the microbial communities associated with slow and fast growing individuals, i.e. small and large larvae of the same age, at ten different developmental stages from 7 to 42 days post hatching (dph).

## Results

### Standard length, myotome height and dry weight of small and large individuals

At each of the 10 sampling times, 5 small (S) and 5 large (L) individuals were sampled. The growth of the cod larvae was described by measurements of standard length, myotome height and dry weight (DW). There were significant differences in the average length, height and weight of small and large individuals at all ten sampling times (p < 0.05), except for myotome height and dry weight for 7 dph larvae. Consequently, the sampled larvae were suitable representatives for slow- and fast-growing individuals, and for evaluating the relationship between composition of the microbiota and growth rate.

**Figure 1:**
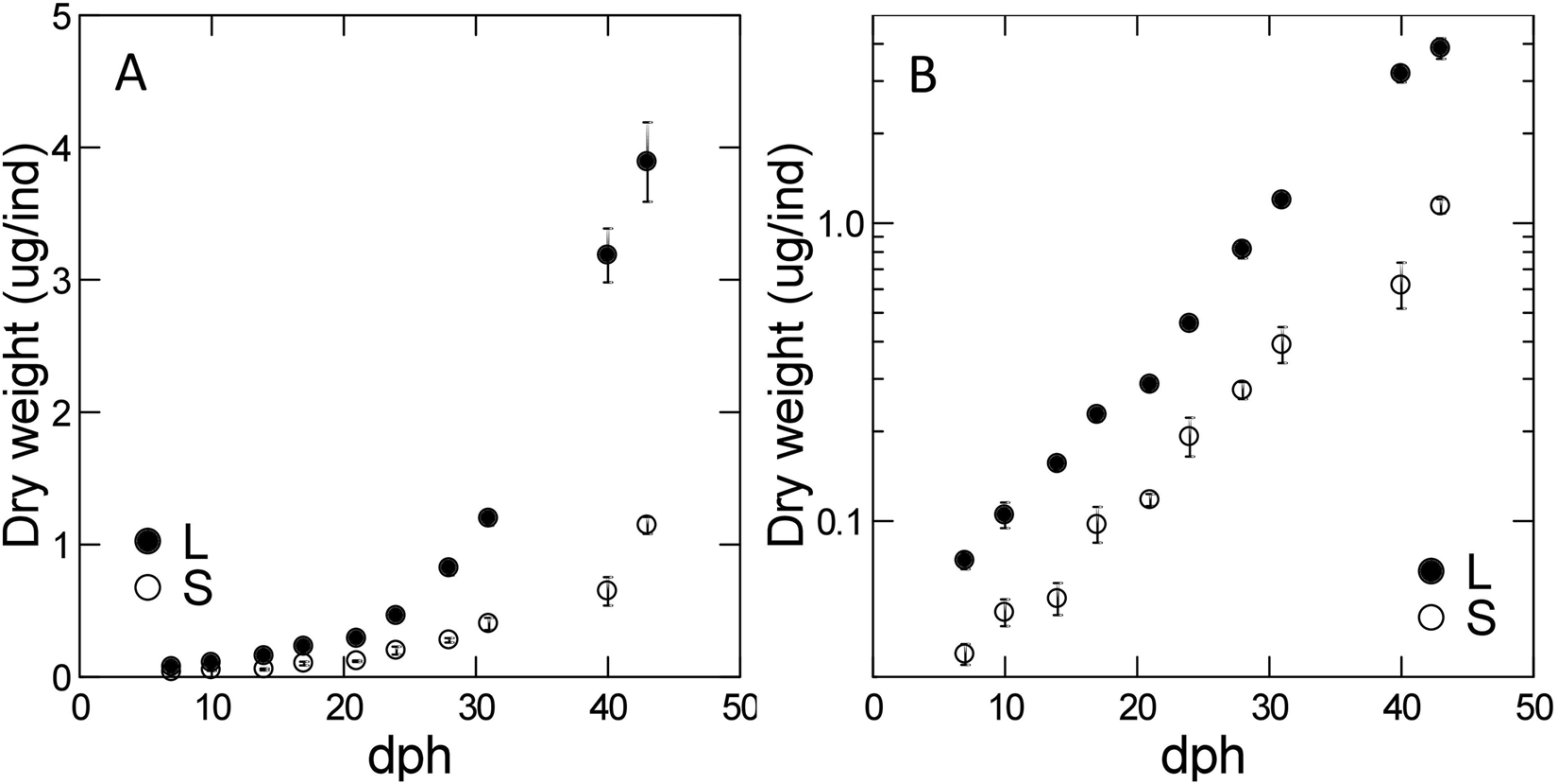
Size development of small (S) and large (L) cod larvae during the experimental period of 42 days presented as average dry weight per individual. (**A**) original data; (**B**) data in logarithmic weight-axis. Error bars are standard error for 5 individuals.

Larval standard length, myotome height and dry weight were closely correlated, and larval dry weight (µg dw/ind.) could be calculated from length and height (mm) by power relationships:

$${\rm{DW}}=(1.126\pm 0.025)\cdot {({\rm{height}})}^{1.892\pm 0.038}$$

$${\rm{DW}}=(0.000549\pm 0.000097)\cdot {({\rm{length}})}^{3.244\pm 0.066}$$

with very high coefficients of determinations (R^2^ ~ 0.99 for both relationships). Both small and large larvae grew exponentially from day 7 to day 42 (Fig. 1). The growth rates of small and large larvae were 0.100 ± 0.009 and 0.112 ± 0.006 day^−1^ (±S.E.), respectively. In general, large larvae were 2.9–4.4 times bigger than small larvae, with increasing difference with age.

### The effect of age and growth rate on the intestinal microbial community of larvae

**Figure 2:**
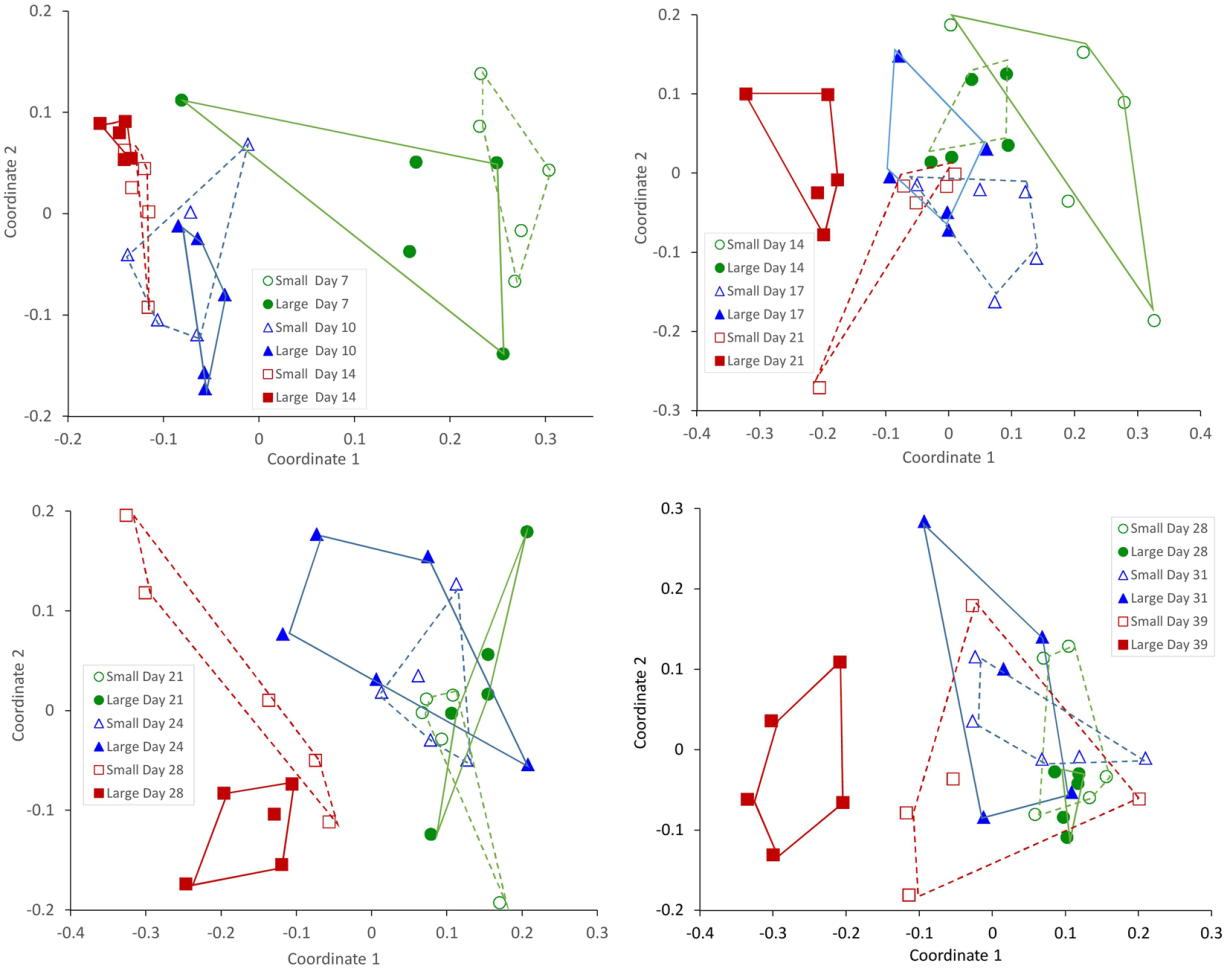
NMDS ordination based on Bray-Curtis similarities for the microbiota of small (S; open symbols) and large (L; filled symbols) larvae for gels 1 to 4. Each symbol represents on individual and the closer symbols are the more similar is their mircrobiota. (**A**) 7 dph (O circle and ● filled circle), 10 dph (△ triangle and ▲ filled triangle), and 14 dph (□ square and ■ filled squared); (**B**) 14 dph (O circle and ● filled circle), 17 dph (△ triangle and ▲ filled triangle), and 21 dph (□ square and ■ filled squared); (**C**) 21 dph (O circle and ● filled circle), 24 (△ triangle and ▲ filled triangle) and 28 dph (□ square and ■ filled squared); (**D**) 28 dph (O circle and ● filled circle), 31dph (△ triangle and ▲ filled triangle), and 39 dph (□ square and ■ filled squared).

In this study we investigated two explanatory variables for the bacterial community composition of larvae: 1) low and high specific growth rate (i.e. small and large larvae of the same age) and 2) age (i.e. dph at 10 different levels). DGGE analysis revealed that there were dynamic changes in the bacterial community composition associated with the larvae as they got older. Community profiles of larvae at early and late stages seemed to be more diverse than those of larvae at intermediate ages (14–28 dph). Ordination by non-metric multidimensional scaling (NMDS) based on Bray-Curtis similarities revealed that the microbiota of small and large individuals appeared different at 14, 21, 28 and 39 dph, and it was a general tendency that the larval microbiota differed between the different age groups (Fig. 2). A two-way PERMANOVA using age and size (categorized as small or large) as the main effects revealed significant effect of age for all five DGGE gels, size had a significant effect for only one gel (Gel2), and the interaction effect between age and size was significant for three of the five DGGE gels (Table 1). The F-values for age were 2.5 higher than for the interaction effect. Due to the significant interaction effects further statistical analysis was performed independently for age and size (i.e. growth rate).

**Table 1:** Results from two-way PERMANOVA analysis of microbiota community composition profiles of larval in the 5 DGGE gels using age (dph) and size (categorized as small or large) as the main effects.

DGGE gel	Age (dph)		Size		Interaction	
	F	P	F	p	F	p
Gel 1 (7–10–14 dph)	11.803	0.0001*	1.023	0.370	0.416	0.161
Gel 2 (14–17–21 dph)	4.478	0.0001*	3.278	0.002*	1.775	0.028*
Gel 3 (21–24–28 dph)	6.804	0.0001*	0.965	0.459	1.742	0.047*
Gel 4 (28–31–39 dph)	5.226	0.0001*	1.437	0.169	2.149	0.015*
Gel 5 (31–39–42 dph)	6.942	0.0001*	1.837	0.065	1.215	0.224

F- and p-values are given for main effects and for the interaction effect between age and size. *Indicate significant results.

### Comparison of the intestinal microbial community of small versus large larvae

**Figure 3:**
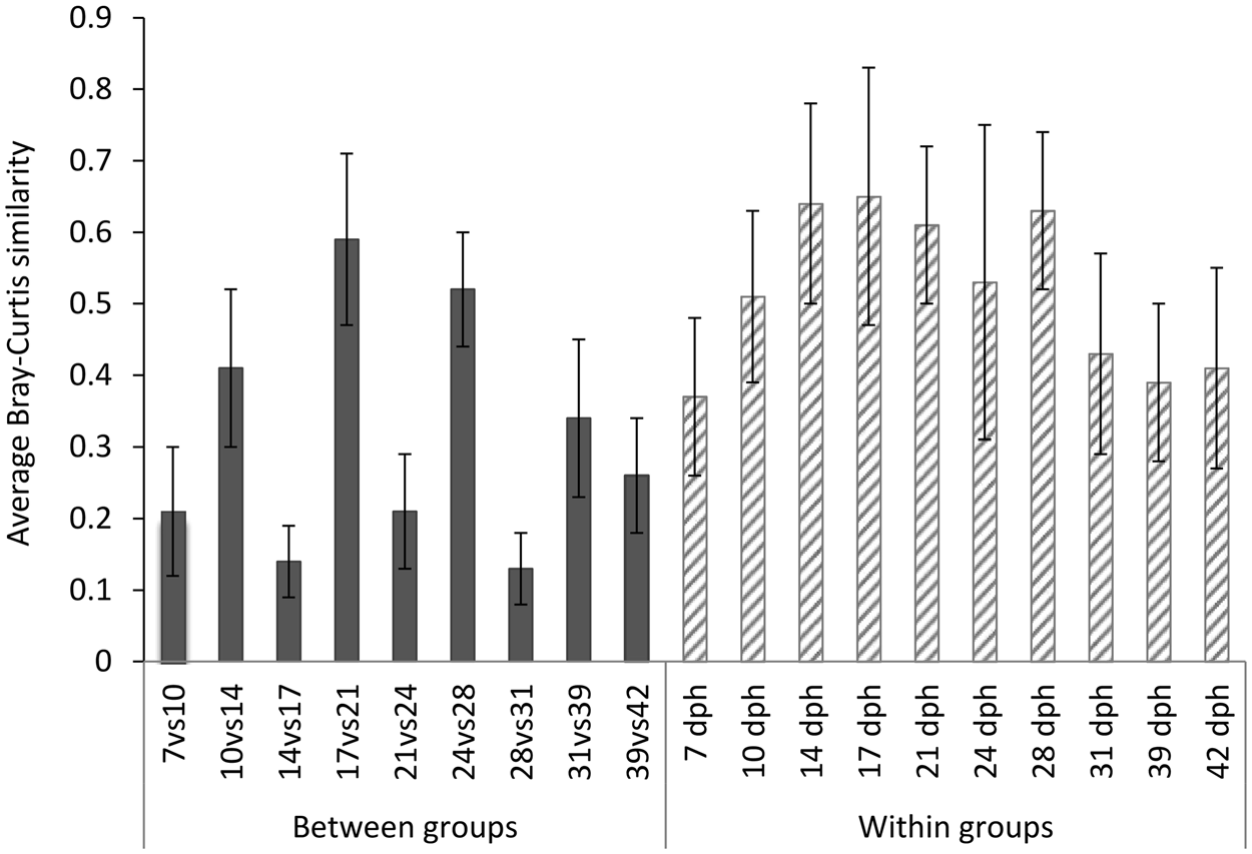
Average Bray-Curtis similarities for comparison of the microbiota of larvae within groups and between age groups (dph: day post hatching). Error bar indicates standard deviation. Number of individuals equal 10 for all days.

Diversity indices (band richness, Shannon’s diversity and evenness) for the bacterial communities of the larvae were calculated from the DGGE banding profiles. The average band richness was significantly higher for small than for large larvae at 14 and 21 dph (p = 0.009, and 0.024, respectively; Table S1). There were no significant differences between small and large individuals in Shannon’s diversity index, except at 14 and 21 dph where it was higher for small than large larvae (p = 0.013 and p = 0.006, respectively). Evenness was significantly higher for large larvae at 24 and 42 dph (p = 0.030 and p = 0.002, respectively). Bray-Curtis similarities indicated that the bacterial community composition among small larvae were more similar at intermediate ages (17–24 dph) than for older and younger larvae (Bray-Curtis similarities of 0.66–0.71 versus 0.35–0.60; Table 2). The similarity in community composition among large larvae of the same age decreased with time, with Bray-Curtis similarities going down from 0.7 to 0.4 (Table 2). The only exception was larvae from 14 dph on Gel 1. These changes in bacterial community composition are visualized by NMDS ordination (Fig. 2). The differences in bacterial community composition are indicated by 1) strong clustering of large and small larvae but limited separation of the two clusters (e.g. 14 dph, Fig. 2A), 2) less clustering within S and L larvae but a clear separation between them (39 dph, Fig. 2D), or 3) a combination of both.

**Table 2:** Average Bray-Curtis similarities (±SE**)** from comparison of the microbiota of equally old small (S) or large (L) larvae (within groups) and comparison of small and large larvae (between groups; S vs L), together with p-values from PERMANOVA for comparison of bacterial community composition of small and large larvae at different ages.

DGGE gel	Age (dph)	Bray- Curtis similarity			p-value
		S vs S	L vs L	S vs L	
1	7	0.431 ± 0.06	0.761 ± 0.06	0.367 ± 0.11	0.470
	10	0.479 ± 0.11	0.513 ± 0.12	0.508 ± 0.13	0.890
	14	0.598 ± 0.14	0.312 ± 0.12	0.606 ± 0.13	0.015*
2	14	0.573 ± 0.16	0.692 ± 0.34	0.572 ± 0.11	0.008*
	17	0.664 ± 0.13	0.671 ± 0.14	0.68 ± 0.09	0.107
	21	0.712 ± 0.31	0.650 ± 0.13	0.612 ± 0.17	0.023*
3	21	0.716 ± 0.13	0.627 ± 0.10	0.665 ± 0.11	0.620
	24	0.699 ± 0.06	0.573 ± 0.08	0.621 ± 0.06	0.290
	28	0.610 ± 0.06	0.747 ± 0.05	0.606 ± 0.04	0.030*
4	28	0.559 ± 0.13	0.721 ± 0.05	0.600 ± 0.11	0.127
	31	0.511 ± 0.09	0.467 ± 0.09	0.468 ± 0.11	0.218
	39	0.414 ± 0.12	0.463 ± 0.01	0.367 ± 0.10	0.007*
5	31	0.551 ± 0.08	0.481 ± 0.09	0.490 ± 0.11	0.26
	39	0.354 ± 0.11	0.376 ± 0.10	0.334 ± 0.10	0.22
	42	0.504 ± 0.13	0.363 ± 0.14	0.387 ± 0.11	0.11

Five S and five L individuals are included at all developmental stages. *Indicate p < 0.05.

One-Way PERMANOVA confirmed significant difference in the bacterial community profiles between small and large individuals at 14, 21, 28 and 39 dph, but not for the other six sampling days (Table 2). Surprisingly, significant differences were normally detected both when between-groups-similarity was high (14, 21 and 28 dph), and when it was low (39 dph). In some cases where the samples have been included on two DGGE gels, the statistical tests are diverging. However, variation in exact denaturing gradients and variable quality among DGGE gels will affect the community profiles, and consequently we put more emphasis on gels with higher resolution.

### Correlations between age and microbial community composition

The highest band richness was observed after onset of first feeding (7 dph) and the band richness varied between 17 and 21 among individuals (Table 3). A general trend of reduced diversity indices was observed up to 28 dph (Table 3). For older larvae, the indices describing diversity of the microbiota increased again. Thus, the diversity of the larval microbiota seemed to be highest at both early and late larval stages. Bray-Curtis similarities were low for comparisons of the larval microbiota community composition between age groups (each group included both small and large larvae; Fig. 3). One-way PERMANOVA analysis revealed significant differences in the community composition of the microbiota between all the 10 age groups (p < 0.0075 and mean p = 0.001, Table S2).

**Table 3:** Average band richness, Shannon index and Evenness for larval DGGE microbial community profiles at different ages (dph). Error bars are SD for 10 individuals.

Age	Band richness	Shannon’s index	Evenness
7 dph	19.3 ± 6.3	2.75 ± 0.34	0.85 ± 0.04
10 dph	18.9 ± 3.0	2.69 ± 0.17	0.79 ± 0.04
14 dph	11.6 ± 3.5	2.04 ± 0.32	0.69 ± 0.03
17 dph	16.6 ± 3.7	2.39 ± 0.25	0.68 ± 0.05
21 dph	16.6 ± 2.5	2.54 ± 0.15	0.69 ± 0.04
24 dph	15.0 ± 4.1	2.23 ± 0.30	0.64 ± 0.06
28 dph	10.4 ± 4.0	2.01 ± 0.38	0.70 ± 0.05
31 dph	21.6 ± 6.4	2.78 ± 0.35	0.78 ± 0.05
39 dph	17.3 ± 4.6	2.62 ± 0.29	0.82 ± 0.04
42 dph	15.1 ± 3.8	2.50 ± 0.30	0.83 ± 0.06

Despite significant differences in the microbiota of cod larvae between all age groups, at 14, 17, 21, 24 and 28 dph two particular DGGE band were strong in the DGGE profiles of almost all individuals (Fig. S1). Both these bands were found to represent the genus *Arcobacter* (Epsilon-proteobacteria).

### Correlations of age and growth rate with microbiota composition of larvae

**Figure 4:**
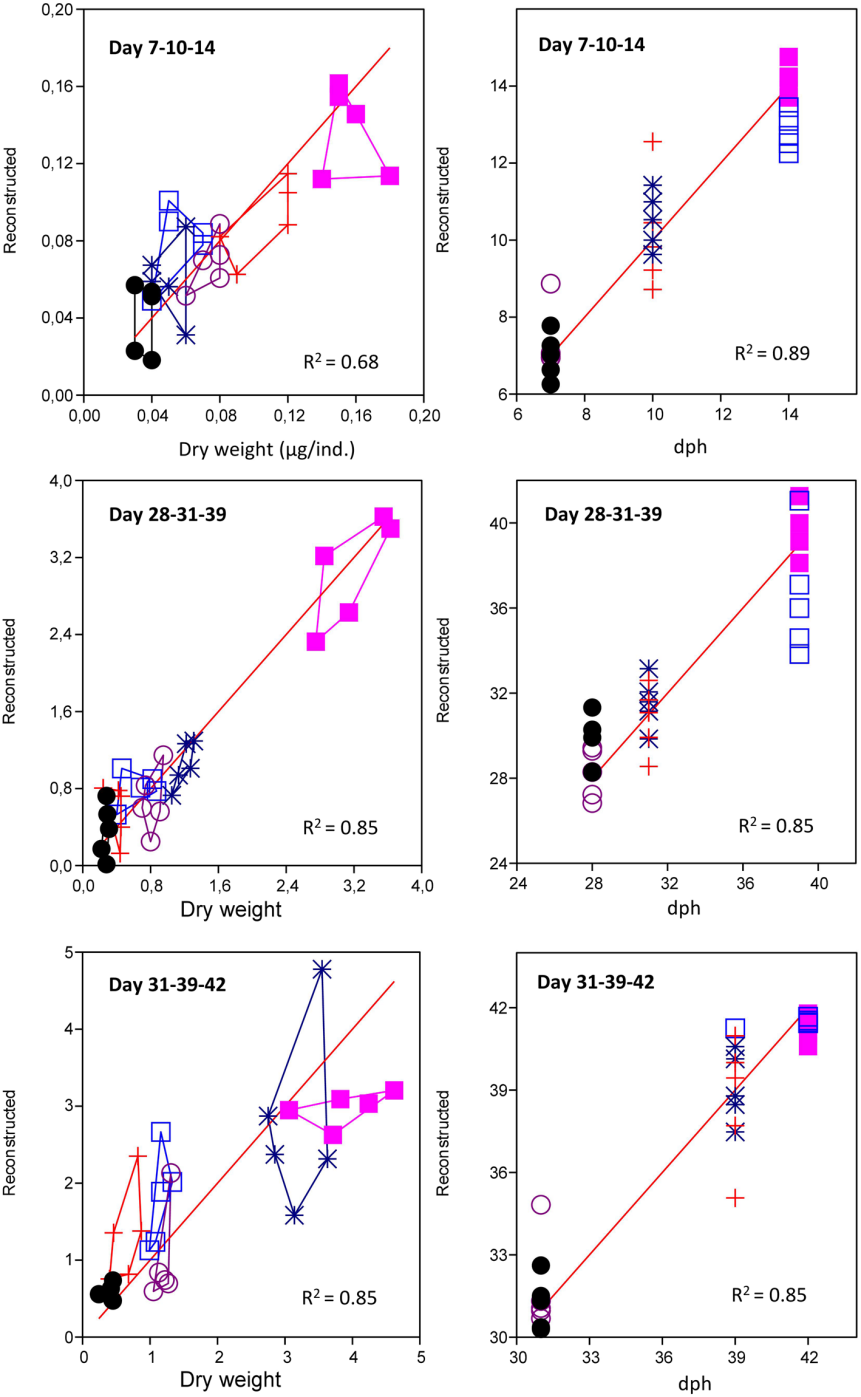
Reconstruction of individual weight (left) and age (right) data of cod larvae based on microbial community composition using factor analysis and environmental regression (CABFAC). dph refers to days post hatching. In each panel the youngest stage is shown by circles, the medium by cross and star, and the oldest by squares. Results for the last two gels are presented in Figure S2.

In the previous sections, we only distinguished between small and large larvae, and used this as a proxy for slow and rapid growing larvae. In the following we use the individual data for size and age in an analysis of larval community composition. The size in terms of dry weight and the age of individual larvae were reconstructed based on their microbial community composition by the use of factor analysis and environmental regression (CABFAC)^17^. The model revealed that both size and age were correlated to intestinal microbial composition of individual larvae (Figs 4 and S2). Whereas age reconstructed the variation in gut microbiota with a high and stable R^2^ throughout the experiment (range 0.83–0.89), this was not the case for size. Interestingly, for the younger stages ( ≤ 21 dph) R^2^ for weight was considerably lower than the one for age (range 0.65–0.68), but for older stages (≥28 dph) R^2^ for weight was equal to the one for age (0.85). Thus, whereas age had stronger correlation to the composition of microbiota than size at young stages, both age and size had similar correlations to the composition of the microbiota of larvae at the older stages.

## Discussion

The intestinal microbiota is important for host health, and different factors may modify and structure the microbiota, including genetic background, developmental stage and environmental factors^4, 18–20^. In this study, the main goal was to investigate potential correlations between the composition of the microbiota and the growth rate of developing Atlantic cod larvae, relative to the changes induced by the age of the fish. Small and large individuals of the same age were used as a proxy of individuals with low or high growth rate, respectively.

Our study showed that the microbial community composition of larvae was significantly different between each sampling day during the 42 days of the experiment (Fig. 3 and Table S2), demonstrating that the composition of the larval microbiota changed considerably with age. The observed changes of the microbiota with age could be explained by changes in the selective pressure inside the host due to the development of the intestinal system^21^. This selection could be caused by both host-microbe and microbe-microbe interactions. The age dependent change of the environments in the larval gut is due to the ontogeny of the digestive tract, implicating huge changes during larval development^11^.

The DGGE profiles of the larval microbiota in the period 10 to 24 dph show that the intestinal microbiota of the larvae during these days changed less than for younger and older stages, and the microbiota was strongly dominated by a few species and with high abundance of *Arcobacter-*related strains (Fig. S1). This corroborates findings in Bakke *et al*.^21^ where microbiota of cod larvae was characterized by pyrosequencing of 16 S rDNA amplicons, but with fewer time points than in the present study. The DGGE community profiles indicated that new bacterial strains were established in the larval microbiota after day 24, leading to the increase in diversity of the microbial community from this day onwards. The high diversity at the first sampling time (7 dph) found in this study could be explained by high load of bacteria through the supply of live food and microalgae^8^ into an un-colonized ecosystem. We also found low Bray-Curtis similarities been individuals at late larval stages. This is in concordance with the findings for developing zebrafish^20^, for which the gut microbiota became more divergent with increasing age.

Forberg *et al*.^10^ showed that size of Atlantic cod larvae was strongly correlated to their intestinal microbiota. However, only larvae sampled at 43 days after hatching were examined in that study. In the present study we found significant differences in the intestinal microbiota of slow and fast growing larvae, i.e. small and large larvae of the same age, for 40% of the age stages studied. In humans and mice important differences in the intestinal microbial communities has been recorded between lean and obese individuals. However, whereas we studied the early life stages of cod larvae, studies with humans and mice were performed with adult stages^6, 7, 22^. It is well established that the microbiota of early life stages is more variable than for adults. Furthermore, fish have a closer interaction between microbiota of the fish and the microbiota in the environments than terrestrial animals, as microbes and fish share the aqueous habitat. The bacteria in the water are actively taken up by marine larvae at rates that are 100X higher than their drinking rate^23^, and through defecation fish re-inoculate the water continuously^11^. This continuous interaction between the microbiota of different individuals kept in the same environment may result in a continuous inter-individual remodulation of the gut microbiota, with a muting of the possible connection between microbiota and growth rate as a consequence. Rearing of fish in individual units is a possible way around this problem. Still, it is interesting to note that the larval microbiota was more correlated to host age than size (in term of dry weight) for younger larval stages (<28 dph) (Fig. 4), which could be due to the fact that size difference between small and large larvae were smaller for the younger than for the older larvae (Fig. 1). Also Bakke *et al*.^21^ demonstrated increased variation between individuals in microbial community structure at older larval stages. In conclusion, the correlation found between size/growth rate and microbiota is consistent with findings from mice, where a positive contribution from the microbiota on energy gain from the diet is documented^7^. An increased energy gain can be due to both increased food intake (appetite)^24^ and improved digestion^6^. However, our experimental design does not allow for a mechanistic evaluation of possible causalities. Testing of this hypothesis could be done by microbiota transplantation studies, similar to those done in mice^6^.

Many factors including internal, external and stochastic factors contribute to shape the intestinal microbiota of the larvae. In the present experiment the fish larvae may have originated from different parents with heterogeneous genetic background, but were exposed to the same environment and rearing conditions. During the rearing period, all the larvae shared this “common garden”, and thus the environment was similar between individuals. As a consequence, possible differences between individuals may have been “diluted” due to this sharing of microbes by small and large larvae in a “common garden”. Because environmental factors such as diet, salinity, and temperature were the same, they could not have contributed to the differences in larval gut microbiota in our study. The observed differences in the community structure of the larval microbiota between small and large individuals could thus have been affected by underlying differences in genetic background among larvae with different parental origin.

In conclusion, there was a strong correlation between age and the composition of the larval microbiota. Moreover, at 4 of 10 developmental stages we detected significant differences in the larval microbiota of slow and fast growing larvae, and size explained as much of the variation in the microbiota as age in the second half of the larval stage. More studies are necessary to test the impacts of genetic background in structuring the bacterial community composition of individuals. However, our data provides support to the hypothesis that larval development may structure the microbiota through a change in selection pressure by fish-microbe and microbe-microbe interactions, and that the composition of the microbiota may influence larval growth rate by improved energy gain. Further studies are needed to test underlying mechanisms.

## Materials and Methods

### Feeding experiment and Sampling

The cod eggs originated from a group spawning at the Norwegian Cod Breeding Centre in Tromsø, Norway. The eggs were disinfected with 400 ppm glutaraldehyde for 10 min^25^ and rinsed before they were incubated in conical tanks containing 34 ppt seawater of 5 °C and with flow rate 3 L h^−1^. Just before hatching the eggs were transferred to a 150 L rearing tank at NTNU Sealab at an initial density of 100 larvae L^−1^. 70% hatch of the eggs was defined as 0 dph (day post hatching). The larvae were reared for six weeks post hatch and fed live feed automatically by a robot. Rotifer was fed to cod larvae in 2 to 8 meals per day during the period from 3 to 24 dph. From 24 to 27 dph the rotifer quantities were reduced and replaced by Artemia nauplii. The live feed was always present in surplus, and live feed addition was based on counts of live feed densities in the tanks. The temperature was 7 °C the first 2 days, and then slowly increased until it reached 12 °C at 11 dph. Thereafter the temperature remained unaltered for the rest of the experiment. The water exchange was gradually increased from 2 volume exchanges per day until it reached 10 per day at 37 dph. Details on experimental conditions are found in Rehberg *et al*.^26^.

All samples were taken from the same tank to avoid tank effects, but replicate tanks^26^ verified that the sampled tank behaved typically. For sampling of larvae a plastic tube was placed at the mean depth in the centre of the tank, and 100–110 individuals were collected by siphoning each sampling day. This sampling affected population development much less than the mortality in the tanks^26^. From these larvae, the 5 biggest and the 5 smallest individuals were sampled for further analysis. Larvae were collected at 7, 10, 14, 17, 21, 24, 28, 31, 39 and 42 dph. The sampling was carried out so that periods just after feeding were avoided. The experiment was approved by the Norwegian “Mattilsynet” and conducted according to Norwegian legislation for experiments with animals and supervised by authorized personnel.

### Measurement of length, height and dry weight of cod larvae

Collected larvae from each sampling day were anesthetized using tricaine methanesulfonate (MS-222, Finquel^®^, Agent Chemical Laboratories Inc, and USA) in cool seawater by placing a beaker containing larvae on ice. The larvae were photographed individually using stereomicroscope (M-80, Leica, Wetzlar, Germany and a DFW-SX900 camera, Sony, Minato, Japan), and measurements of standard length and myotome height was done using the ImageJ software. After photography larvae were rinsed 3 times in sterile milli-Q water to remove salts, and then transferred individually to Eppendorf tubes and frozen at −20 °C. To obtain dry weight of the larvae they were lyophilized for 24 h and weighed to the nearest 0.001 mg (Metler MT5).

### DNA extraction, PCR, and DGGE

DNA was extracted from single larvae using PowerSoil^®^ DNA Isolation Kit (MoBio) according to the manufacturer’s instructions. Prior to DNA extraction the larvae were homogenized using a glass rod. An aliquot of 2 µl DNA extract was analysed with a NanoDrop ND-1000 spectrophotometer (Thermo Fisher Scientific) to determine the concentration of extracted DNA.

The DNA extracts were used as templates for amplification of the variable V3 region of the bacterial 16 S rRNA gene by using a nested PCR protocol. The first PCR was with universal Bacteria specific primers to avoid co-amplification of eukaryotic DNA, and the second PCR was with primers suitable for DGGE. Details are described in Bakke *et al*.^27^. As a template in the external PCR, approximately 20 ng total DNA was used, and 1 µl of external PCR reactions product was used as the templates in the internal PCR. PCR products were analysed by agarose gel electrophoresis. Twenty cycles were used for both the external and internal PCR.

DGGE analysis was performed using an INGENY phorU system (Ingeny) as described in Bakke *et al*.^26^ using 8% polyacryalmide gels with a denaturing gradient of 35 to 55% (100% correspond to 7 M urea and 40% formamide). After electrophoresis the DGGE gels were stained with SYBR® Gold (Invitrogen) for 1 hour at room temperature and photographed under UV in a GenBox geldoc system (Syngene). A maximum of 30 samples could be analysed on each DGGE gel, and we avoided between-gel comparisons of DGGE profiles. The samples for some sampling days (dph 14, 21, 28, 31 and 39) were therefore run twice to allow comparison of all “neighbouring” sampling days. In total 5 DGGE gels were run, each including 5 small and 5 large individuals from three sampling times: Gel 1 for 7, 10 and 14 dph larvae; Gel 2 for 14, 17 and 21 dph larvae; Gel 3 for 21, 24 and 28 dph larvae; Gel 4 for 28, 31 and 39 dph larvae and Gel 5 for 31, 39 and 42 dph larvae. S and L refer to small and large larvae, respectively. DNA sequencing of DGGE bands were performed as described in Bakke *et al*.^28^ by first removing a part of the band with the tip of a pipette and the sequencing the reamplified band by Sanger sequencing.

### Analysis of gel images and statistical analysis

The DGGE gel images were analysed using the image analysis software Gel2k v.1.2.0.6, curtesy of Svein Norland, University of Bergen, Norway (http://folk.uib.no/nimsn/gel2k/), for converting banding patterns to desi-metric profiles, band identification and calculation of peak area of each band. As an output this gives a sample-peak area matrix. The peak areas for each sample were normalized to percent of the total peak area, and the normalized matrix was used for further statistical analyses. To characterize the microbial communities of the samples (α-diversity) various diversity indices were calculated. To visually compare DGGE-profiles between samples (β-diversity) we used ordination by non-metric multidimensional scaling (NMDS) based on Bray-Curtis similarities calculated for all combinations of samples^29^. NMDS is well suited for such data as it makes no assumptions of normality and linearity, and can represent a large part of the variation in a dataset in two dimensions as it preserves rank and not absolute similarities. To test for differences in α-diversity indices between groups of samples we used ANOVA, and for statistical analysis of differences in bacterial community composition (β-diversity) we used one-way and two-way permutational multivariate analysis of variance (PERMANOVA) based on Bray-Curtis similarities^30^. All statistical analyses were done with PAST version 2.17^31^, except for larval length, height and weight data which were analysed with SYSTAT (v 13, SYSTAT Software Inc.).

## Electronic supplementary material

Supplementary material
