# Supplementary material for: Correlations of age and growth rate with microbiota composition in Atlantic cod (Gadus morhua) larvae
Source: Sci Rep. 2017 Aug 17;7:8611. doi: 10.1038/s41598-017-09073-9 (PMC5561051; doi:10.1038/s41598-017-09073-9)
Supplement: Supplementary file 1 — Supplementary material [file 41598_2017_9073_MOESM1_ESM.doc]

Correlations of age and growth rate with microbiota composition in Atlantic cod (*Gadus morhua*) larvae

Ly T T Trinh^1, 2^, Ingrid Bakke^1^, Olav Vadstein^1 *^

^1^ Department of Biotechnology, NTNU Norwegian University of Science and Technology, N7491 Trondheim, Norway

^2^ School of Biotechnology, International University, Vietnam National University, Quarter 6, Linh Trung ward, Thu Duc District, HoChiMinh City, Vietnam

^*^Corresponding author: [olav.vadstein@ntnu.no](mailto:olav.vadstein@ntnu.no)

**SUPPLEMENTARY INFORMATION**

**Table S1:** ANOVA analysis for testing the differences in diversity indices between large (L) and small (S) larvae at different ages. *: p<0.05

|  |  | **ANOVA P-value** | | |
| --- | --- | --- | --- | --- |
|  |  | **Band richness** | **Shannon index** | **Evenness** |
| **Between groups** | **7S vs 7L** | 0.323 | 0.576 | 0.670 |
|  | **10S vs 10L** | 0.769 | 0.646 | 0.710 |
|  | **14S vs 14L** | 0.009* | 0.013* | 0.130 |
|  | **17S vs 17L** | 0.640 | 0.622 | 0.970 |
|  | **21S vs 21L** | 0.024* | 0.006* | 0.780 |
|  | **24S vs 24L** | 0.239 | 0.823 | 0.030* |
|  | **28S vs 28L** | 0.420 | 0.390 | 0.490 |
|  | **31S vs 31L** | 0.850 | 0.850 | 0.920 |
|  | **39S vs 39L** | 0.340 | 0.220 | 0.768 |
|  | **42S vs 42L** | 0.59 | 0.22 | 0.002* |

**Table S2:** p-values from a One-way PERMANOVA test of differences in larval microbiota between “neighbouring” sampling days (one age group and the age one level older).

| Groups | p-value |
| --- | --- |
| 7 dph vs 10 dph | 0.0001 |
| 10 dph vs 14 dph | 0.0003 |
| 14 dph vs 17 dph | 0.0001 |
| 17 dph vs 21 dph | 0.0075 |
| 21 dph vs 24 dph | 0.0001 |
| 24 dph vs 28 dph | 0.0001 |
| 28 dph vs 31 dph | 0.0001 |
| 31 dph vs 39 dph | 0.0018 |
| 39 dph vs 42 dph | 0.0001 |


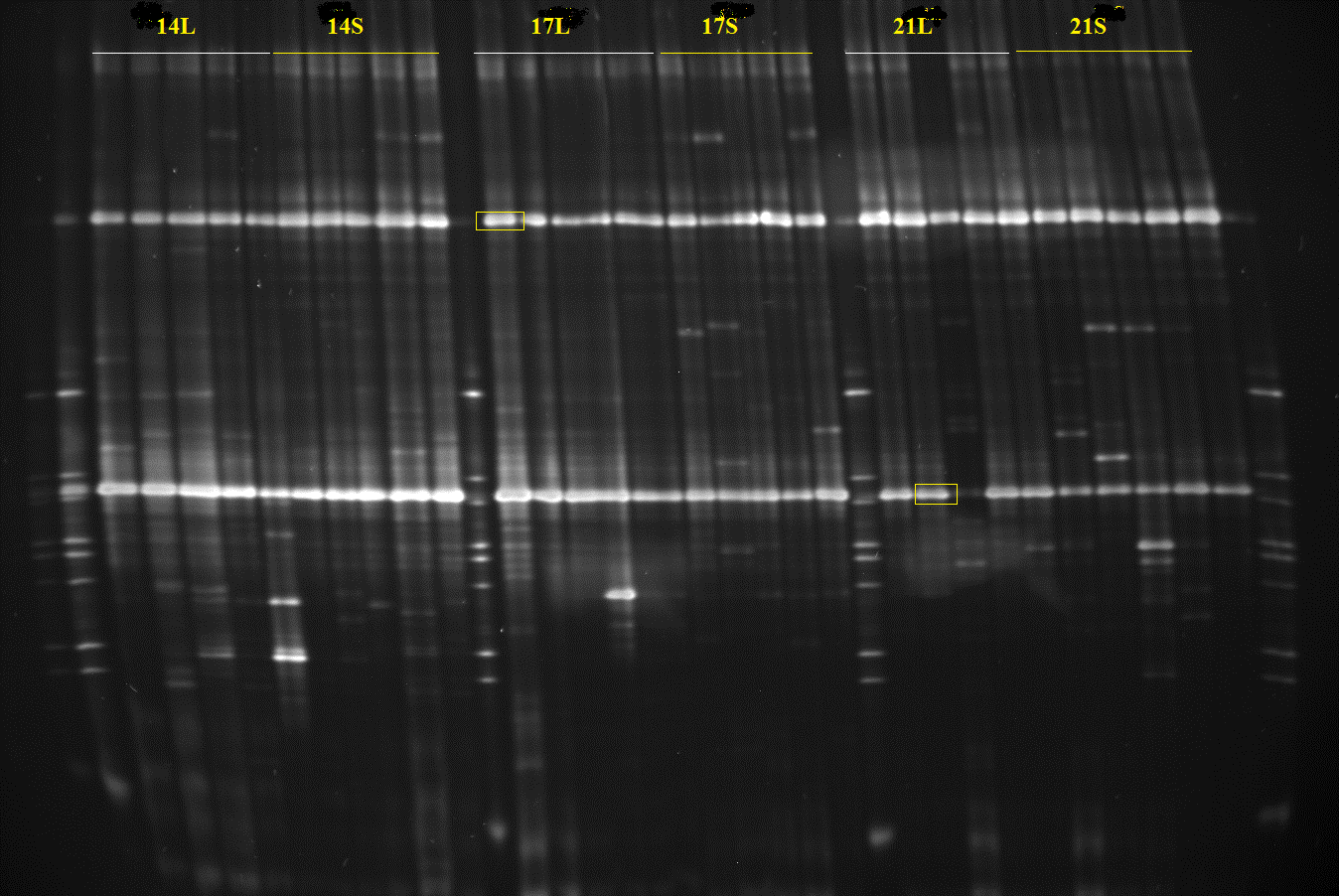

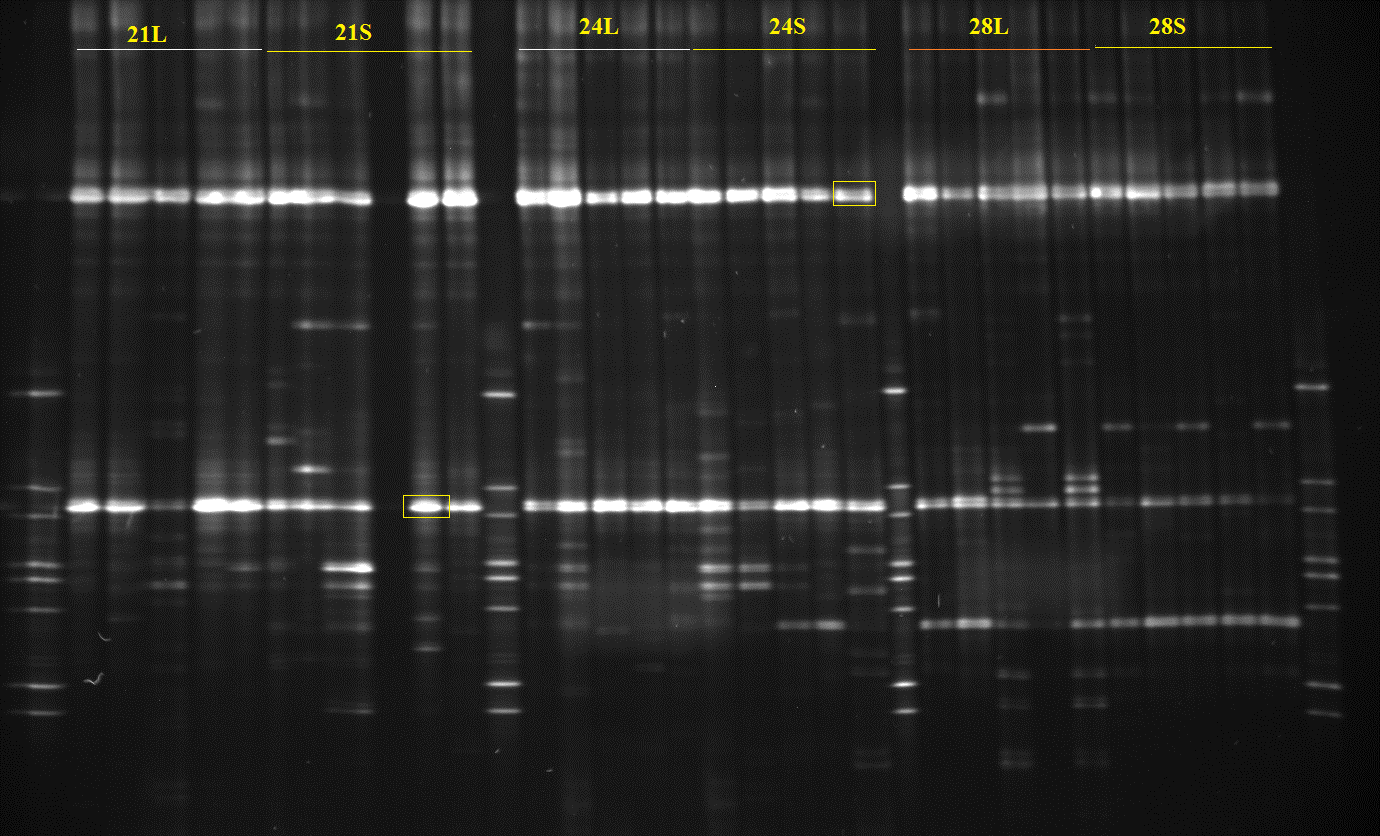


**Figure S1:** Denaturing gradient gel electrophoresis (DGGE) profiles for PCR-amplified 16S rDNA fragments from microbial communities of large (L) and small (S) cod larvae. The marked bands are those which were sequenced and assigned to be *Arcobacter* species.

Dry weight (µg/ind.)

R^2^ = 0.65

**Day 14-17-21**

dph

R^2^ = 0.83

**Day 14-17-21**

Dry weight (µg/ind.)

R^2^ = 0.68

**Day 7-10-14**

**Day 7-10-14**

R^2^ = 0.89

dph

**Figure S2**: Reconstruction of individual weight and age data of cod larvae based on intestinal microbial community composition using factor analysis and environmental regression (CABFAC). dph refers to days post hatching. Data for remaining three gels are shown in the original paper.
